# Genetic relationship study of some *Vicia* species by FISH and total seed storage protein patterns

**DOI:** 10.1186/s43141-020-00054-6

**Published:** 2020-07-31

**Authors:** Hoda B. M. Ali, Samira A. Osman

**Affiliations:** Genetics and Cytology Department, Genetic Engineering and Biotechnology Research Division, National Research Centre, P.O, Giza, 12622 Egypt

**Keywords:** *Vicia*, FISH, rDNA, SDS-PAGE, Genetic relationships

## Abstract

**Background:**

Genus *Vicia* is a member of family Fabaceae and comprises 180 to 210 species. The most important species is faba bean (*Vicia faba*) which is still one of the most favourable grain legumes over all the world. The genus contains some additional food crops and a number of forage plants and some other weedy strains such as *Vicia angustifolia* and *Vicia cordata*. The aim of the present investigation is to elucidate the phylogenetic relationships among four *Vicia* species, two species (*Vicia angustifolia* L. ssp. Angustifolia (2n = 12) and *Vicia cordata* wulfen ex Hoppe (2n = 10)) belong to section *Vicia*, *Vicia dalmatica* A. Kern (2n = 12, section Cracca), and *Vicia johannis* tamamsch (2n = 14, section Faba).

**Results:**

Two tools have been applied to identify the genetic relationships among the examined species, double fluorescence in situ hybridization (FISH) has been used to localize the sites of 5S and 45S rDNA, and sodium dodecyl sulfate-poly acrylamide gel electrophoretic (SDS-PAGE) patterns of total seed storage protein fractions. Double FISH experiment has not shown any variation in the loci number, but the positions along the chromosomes were different; both *Vicia johannis* and *Vicia dalmatica* exhibited the same interstitial 45S rRNA gene loci, while *Vicia angustifolia* and *Vicia cordata* have shown single large stretched 45S rRNA loci almost at the terminal region of the shortest chromosome. It could be concluded from the similarity matrix among the *Vicia* species as computed according to Jaccard coefficient from the SDS-PAGE, that *V*. *cordata* is similar to *V*. *angustifolia* and *V*. *dalmatica* by a percentage of 73 and 69%, respectively, and the most related species to *V*. *johannis* is *V*. *dalmatica* (~ 64%).

**Conclusion:**

FISH and SDS-PAGE of the total seed storage proteins together reflected the similar genetic relationship among the studied species as fellows, *V*. *angustifolia* is more related to *V*. *cordata* then comes *V*. *dalmatica* and then *V*. *johannis* which is at a distal position from the other species.

## Background

*Vicia* is a member of the tribe Vicieae (family Fabaceae) and considered a medium-sized genus; the number of species can be estimated as 180 to 210 [1]. This genus has a considerable economic importance species especially *Vicia faba* and *Vicia ervilia*, since faba bean is still one of the most favourable grain legumes in the moderate regions of all the world. In addition, the genus contains some additional minor food crops and more than a dozen forage plants, such as the most important common vetch, *V*. *sativa*, other species have developed as weedy strains (*Vicia angustifolia* and *Vicia cordata*). The genus is distributed almost over all the world mainly from Asia, Europe, and North America to moderate regions of South America and tropical Africa and mainly located in Mediterranean and Irano-Turanian regions [2, 3]

*Vicia* species were an excellent objection for karyological, cytogenetical, and molecular-genetic studies, especially the very large chromosomes of *V*. *faba* which are suitable for such investigations. The karyological characters of this genus exhibit slight variability, especially in regard to the morphology of chromosomes and the DNA content of the genome. The basic chromosome numbers in genus *Vicia* are X = 5, 6, and 7, and the great majority of the species are diploid and only a small proportion of 4x and even 6x cytotypes have been observed. However, it has been found in a few cases supernumerary chromosomes (B-chromosomes) in cytotypes of *Vicia ohwiana* with 2n = 12 + (0-2B) and *Vicia alpestris* with 2n = 28 + (0-1B), evolutionary implication of such chromosomal deviations is still unknown [4–7].

Fluorescence in situ hybridization investigations consuming rDNA genes as probes have been used in most of plant species [8–11]. The ribosomal DNA repeat units occur at one or more rRNA gene loci in the genome, the 18S–5.8S–25S rDNA scattered in tandem arrays in one or more chromosomal regions known as a secondary constriction or nucleolar organizing regions (NORs), whereas 5S rDNA is usually independent scattered in one or several regions [8, 12–14]. Therefore, rDNA-FISH has been used as an excellent tool to find out the chromosomal evolution within and between related species by recognizing and understanding the chromosomal organization and analysing the genetic relationships. Application of double or multiple probe FISH enables the discrimination of the homologous chromosomes and explores the structural genome changes among many species. It helps to clarify genetic maps and to assign linkage groups to physically marked chromosomes as well [15–18]. Florescence in situ hybridization has been applied in different *Vicia* species [19–25].

Isozymes systems and sodium dodecyl sulfate polyacrylamide gel electrophoresis (SDS-PAGE) as molecular marker tool still efficient for germplasm characterization and had been successfully used to evaluate the genetic diversity. Seed storage protein electrophoresis as an additional SDS-PAGE tool has been applied to elucidate and trace back the evolution of numerous groups of plant species and genera [26–32]. The proteins in the seeds of legumes are ranging from about 20% in pea (*Pisum sativum* L.) and beans (*Phaseolus* spp.), increasing up to 40% in soybean (*Glycine max* (L.) Merr.) and lupin (*Lupinus* spp.) [33].

Seed storage proteins have been studied in several plant species for providing appropriate biological system for the evolution studies. Their patterns could be used to elucidate the degree of diversity between the different species or to discriminate the individuals within the same species [34–38], due to their stability during the course of evolution and are slightly affected by environmental conditions and seasonal changes [39, 40].

The main goal of the present study was to locate the sites of 5S and 45S rDNA as probes by fluorescence in situ hybridization tool on the metaphase chromosomes of four *Vicia* species (*Vicia angustifolia* L. ssp. angustifolia, *Vicia cordata* wulfen ex Hoppe, *Vicia dalmatica* A. Kern, and *Vicia johannis* tamamsch) and by SDS-electrophoretic patterns of total seed storage protein fractions to find out the relationships among them.

## Methods

### Plant material

Four *Vicia* species were obtained from the germplasm collection (Genebank for agricultural and horticultural crops) of the Institute of Plant Genetics and Crop Plant Research (IPK), Gatersleben, Germany. *Vicia johannis* tamamsch (accession no. VIC NAR 46/83) belongs to section Faba with 2n = 14 chromosomes, and two species with 2n = 12, *Vicia angustifolia* L. ssp. angustifolia (accession no. VIC 1178) belongs to section Vicia and *Vicia dalmatica* A. Kern (accession no. VIC 38/93) belongs to section Cracca, while the fourth one, *Vicia cordata* wulfen ex Hoppe (accession no. VIC 453) has 2n = 10 chromosomes and belongs to section Vicia.

### Chromosome preparation

Chromosome preparations from root tips and FISH were done according to [41] with minor modifications. Seeds were sown on two layers of moistened filter paper in a petri dish and kept in the dark at 25 °C for 2 days. The young germinated root tips were cut and treated with 0.02% aqueous 8-hydroxyquinoline for 3 h at 15 °C and then washed three times with sterile water before fixation in freshly prepared chloroform-acetic acid-ethanol (6:3:1) then in acetic acid-ethanol (1:3) and stored in ethanol 70%.

### Fluorescence in situ hybridization

The *A*. *thaliana* BAC clone T15P10 (AF167571) bearing the 45S rDNA sequence was labelled with digoxigenin by nick translation, and the 5S rDNA probe was amplified from genomic DNA of *A*. *thaliana* and labelled with biotin by PCR with primers specific for the coding region [42]. The biotinylated 5S rDNA was detected by avidin~Texas Red (Vector Laboratories) and amplified by biotinylated goat anti-avidin (Vector Laboratories) and avidin~Texas Red. Digoxigenin-labelled probes were detected by mouse anti-digoxigenin (Jackson ImmunoResearch Laboratories) and goat anti-mouse antibodies conjugated with Alexa 488 (Molecular Probes). The chromosomes were counterstained with 4′,6-diamidin-2-phenylindol (DAPI, 2 μg/ml). The images were captured with a Zeiss Axioplan 2 epifluorescence microscope equipped with a Spot 2e CCD camera. Images were pseudo-coloured and merged using Adobe Photoshop CS software (Adobe).

**Fig. 1:**
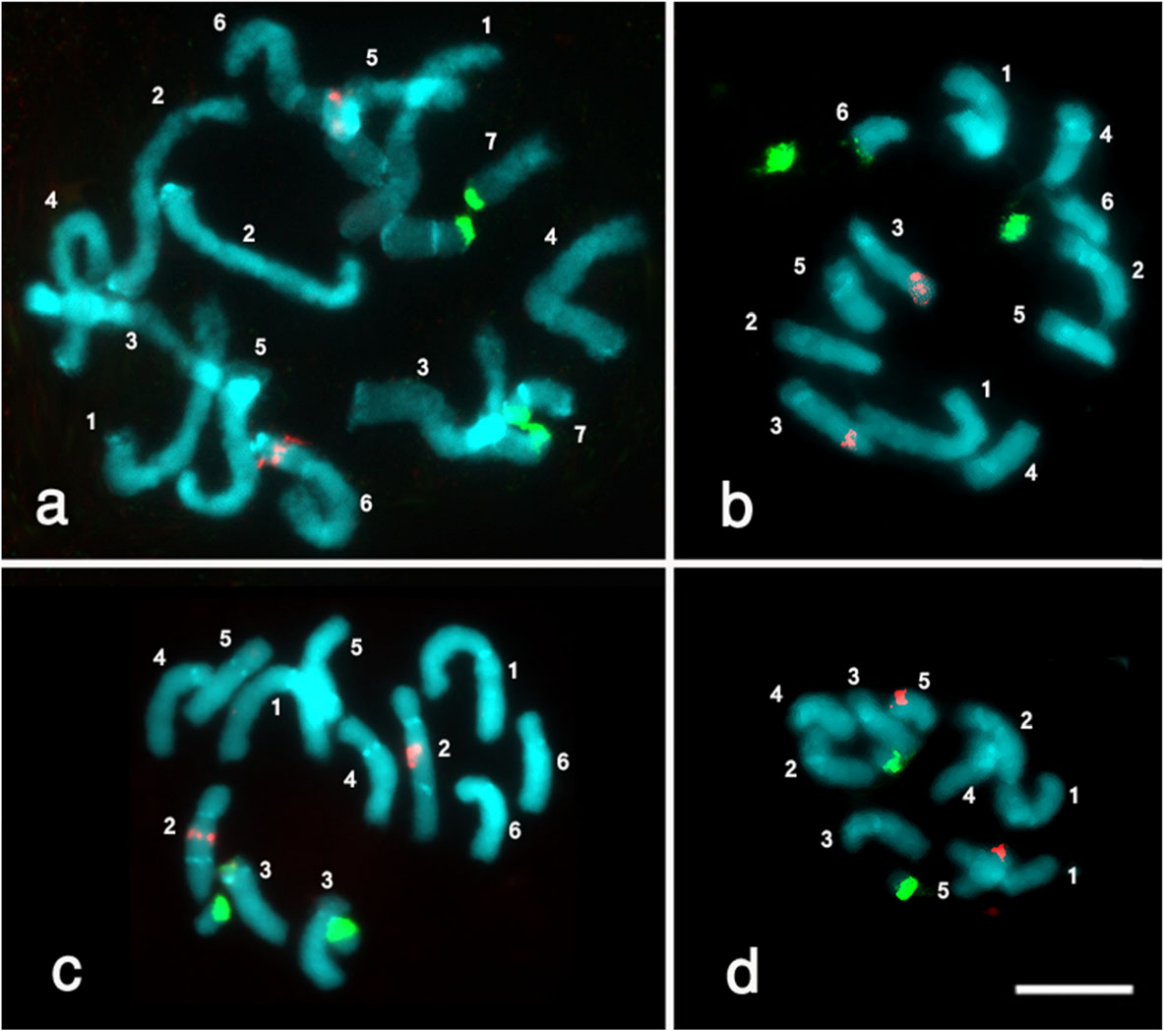
Mitotic metaphase chromosomes of four *Vicia* species after FISH with rDNA probes; 18S-25S rDNA probe was detected by FITC (green signals) and 5S rDNA probe by Texas red (red signals). The chromosomes were counterstained with DAPI. **a***Vicia johannis* (2n = 14). **b***Vicia angustifolia* (2n = 12). **c***Vicia dalmatica* 2n = 12. **d***Vicia cordata* (2n = 10). Bar = 5.0 μm

The classification and numbering of the mitotic chromosomes (Fig. 1) have been done manually for each species from several images which are taken by the microscope. By magnifying the chromosome images in Adobe Photoshop CS software (Adobe) to enlarge the image to the size in which the difference in chromosome size could be clarified, and then arranging the number of these chromosomes according to their decreasing in the size, taking in account the homologous chromosomes which bear the 45S and 5S rRNA genes.

### Proteins profile using SDS-PAGE

Sodium dodecyl sulfate polyacrylamide gel electrophoresis (SDS-PAGE) was performed according to the method of [43] as modified by [44]. Seed storage proteins (total proteins) were extracted from seeds of different *Vicia* species. The marker of used protein was BLUltra Prestained Protein Ladder (GeneDirex, cat no. PM001-0500). In this method, 10% protein separating gel were used. Protein fractionations were performed exclusively on vertical slab gel (19.8 cm × 26.8 cm × 0.2 cm) using the electrophoresis apparatus manufactured by Cleaver, UK. The images were captured by a digital camera (Sony, Japan) and transferred directly to the computer; then, the protein bands were analysed by Total Lab programme to find out the molecular weight of each band, subsequently compare the presence and absence of the band among studied species and these data were imported in MVSP (Multi-Variant Statistical Package [45]) to find the similarity matrix and dendrogram (unweighted pair group method with arithmetic (UPGMA), using Jaccardʼs coefficient) which reflect the relationships among the studied species.

## Results

### rDNA loci

Chromosome number of the analysed species is *Vicia johannis* (2n = 14), *Vicia angustifolia* (2n = 12), *Vicia dalmatica* (2n = 12), and *Vicia cordata* (2n = 10). Despite double FISH experiment using 45S and 5S rDNA probes has not shown any variation in the number of loci, where one 45S rRNA and one 5S rRNA gene loci were detected in the metaphase chromosomes of each of studied species, but the positions along the chromosomes were different, both *Vicia johannis* and *Vicia dalmatica* exhibited the same interstitial 45S rRNA gene loci, while *Vicia angustifolia* and *Vicia cordata* have shown single large stretched 45S rRNA loci almost at the terminal region of the shortest chromosome (Fig. 1). These results have been discussed as follows:

*Vicia johannis* (2n = 14, section Faba) has bigger chromosomes comparing with the other three species, one stretched interstitial 45S rRNA gene site was observed in this species on a smallest chromosome pair (no. 7), which is distinguished with a big satellite, whereas single interstitial 5S rDNA gene site was detected on the short arm of chromosome pair no. 6 (Fig. 1a).

*Vicia angustifolia* (2n = 12, section Vicia), single large 45S rDNA site, stretched and predominantly located in the terminal region of the smallest chromosome pair (no. 6), while 5S rDNA site was located at subterminal position on the short arm of chromosome pair no. 3 (Fig. 1b).

*Vicia dalmatica* (2n = 12, section Cracca) has one large stretched interstitial 45S rDNA site was located on the long arm of middle-sized chromosome pair (no. 3), while the 5S rDNA site was at proximal position on the long arm of large chromosome pair no. 2 (Fig. 1c).

*Vicia cordata* has the lowest chromosome number among the studied species (2n = 10, section Vicia) and is characterized with single terminal 45S rDNA site, and 5S rDNA sites were located separately on the opposite arms of the smallest chromosome pair no. 5 (Fig. 1d).

### SDS-electrophoretic patterns

**Fig. 2:**
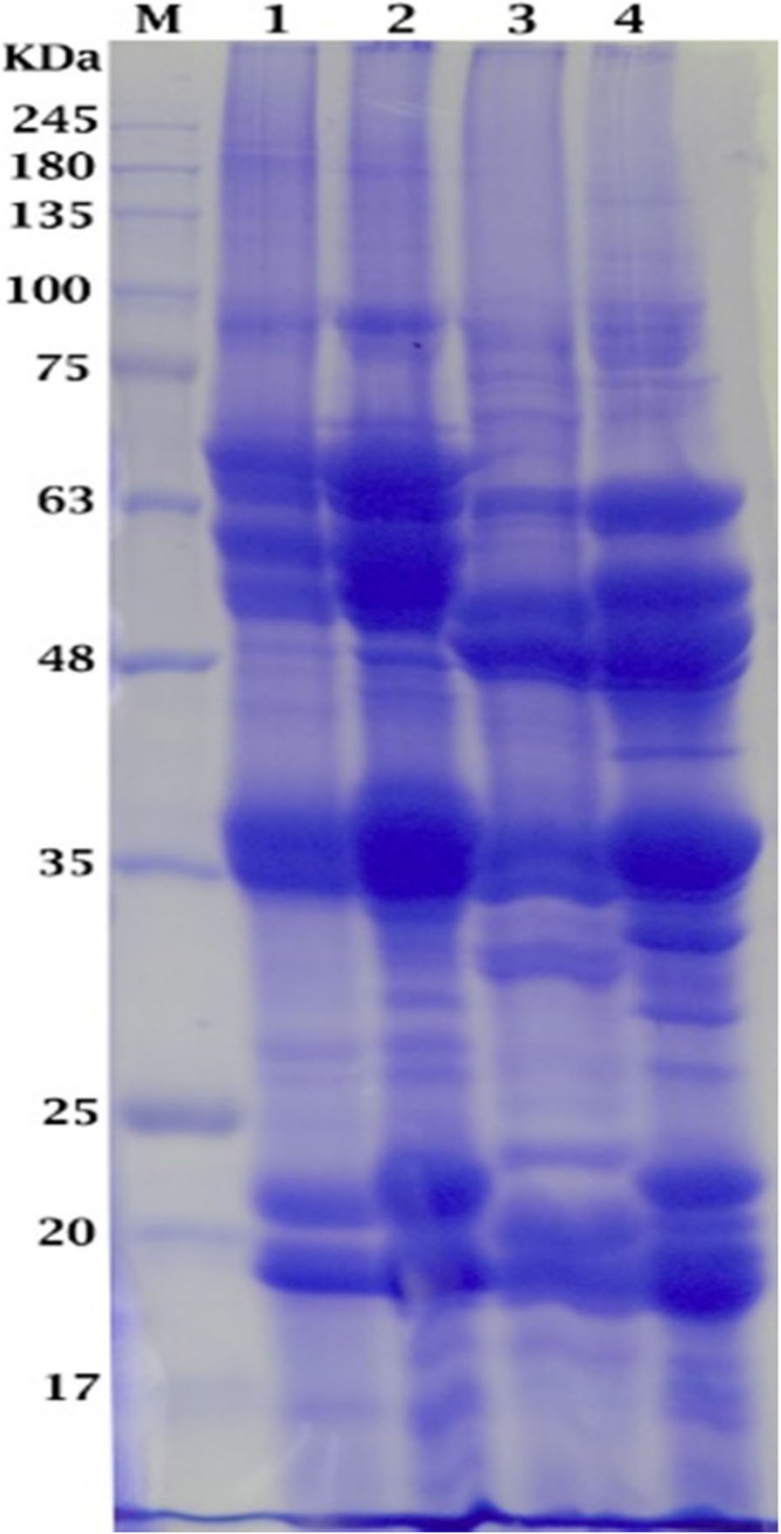
Electrophoretic patterns of the total seed storage protiens in four *Vicia* species. 1, *Vicia angustifolia* L. ssp. Angustifolia. 2, *Vicia cordata wulfen* ex Hoppe. 3, *Vicia dalmatica* A. Kern. 4, *Vicia johannis* tamamsch   M, Protein marker

SDS-electrophoretic pattern of total seed storage protein fractions in the studied *Vicia* species reflected a total number of 44 bands with molecular weight ranging from 210 to 16 Kilo Daltons (KD). The distribution of seed storage protein bands in the four *Vicia* species is shown in Table 1 and Fig. 2. A maximum number of 35 bands were detected in *Vicia cordata*, whereas the minimum number was 29 bands in *Vicia angustifolia*. Although the number of detected bands in *Vicia dalmatica* and *Vicia johannis* was 31 and 30 bands, respectively, the monomorphic bands were sixteen at Mw 175, 100, 90, 80, 67, 63, 49, 47, 44, 36, 35, 31, 27, 19, 18, and 17 KD.

**Table 1:** Approximate molecular weight and intensity of total seed storage protein bands in four *Vicia* species

Band number	MW (KDa)	***Vicia angustifolia***	***Vicia cordata***	***Vicia dalmatica***	***Vicia johannis***
**1**	**210**	+	+	-	-
**2**	**175**	+	+	+	+
**3**	**150**	-	-	-	**+**
**4**	**140**	-	-	-	**+**
**5**	**130**	**+**	**+**	-	-
**6**	**120**	**+**	-	-	-
**7**	**115**	-	**+**	**+**	-
**8**	**110**	-	-	-	**+**
**9**	**100**	+	+	+	+
**10**	**90**	++	++	+	+
**11**	**80**	+	+	+	+
**12**	**75**	-	-	**+**	**+**
**13**	**71**	**-**	+	+	+
**14**	**67**	+	+	+	+
**15**	**65**	+++	+++	+	**-**
**16**	**63**	+++	+++	++	+++
**17**	**62**	-	-	**+**	-
**18**	**60**	+++	+++	+	**-**
**19**	**56**	+++	+++	-	+++
**20**	**54**	+++	+++	+	**-**
**21**	**49**	+	+	+++	+++
**22**	**47**	+	+	+++	+++
**23**	**46**	-	**+**	-	-
**24**	**44**	+	+	+	+
**25**	**41**	-	-	**+**	**++**
**26**	**39**	+	++	+	**-**
**27**	**37**	-	**++**	**+**	-
**28**	**36**	+++	+++	+++	+++
**29**	**35**	+++	+++	+++	+++
**30**	**33**	**-**	+	+	+
**31**	**32**	**-**	+	++	++
**32**	**31**	+	+	++	+
**33**	**30**	+	+	**-**	++
**34**	**28**	**+**	**+**	-	-
**35**	**27**	+	+	+	+
**36**	**25**	+	+	+	**-**
**37**	**23**	-	**++**	**++**	-
**38**	**22**	+++	+++	**-**	+++
**39**	**21**	-	-	-	**++**
**40**	**20**	++	**-**	++	++
**41**	**19**	+++	+++	+++	+++
**42**	**18**	+	+	+	+
**43**	**17**	+	+	+	+
**44**	**16**	-	**+**	-	**+**
**Total number of bands**		**29**	**35**	**31**	**30**

*Vicia angustifolia* exhibited the lowest number of bands (29 bands) among the examined species. The largest size band was at Mw 210 KD and the smallest band was at 17 KD. This species was characterized by having one +ve unique band at Mw 120 KD, and three –ve unique bands at Mw 71, 33, and 32 KD. This species shared with *Vicia cordata* in 2 bands (130 and 28 KD).

The maximum number of bands (35 bands) was detected in *Vicia cordata*. The largest-size band was at Mw 210 KD and the smallest band was at 16 KD. This species was characterized by the presence of one +ve unique band at 46 KD, and one –ve unique band at 20 KD. This species shared with *Vicia angustifolia* in 2 bands (130 and 28 KD), *Vicia dalmatica* in 3 bands (115, 37, and 23 KD), and *Vicia johannis* in one band at 16 KD.

*Vicia dalmatica* had a total number of 31 bands. The largest size band was observed at Mw 175 KD and the smallest band was detected at Mw 17 KD. It was characterized by the presence of one unique band at 62 KD. It shared with *Vicia cordata* in 3 bands (115, 37, and 23 KD) and *Vicia johannis* in 2 bands (75 and 41 KD).

In *Vicia johannis*, a total number of 30 bands was detected, the highest one at 175 KD, whereas the lowest one was at 16 KD. This species was characterized by the presence of four unique bands at Mw 150, 140, 110, and 21 KD.

**Fig. 3:**
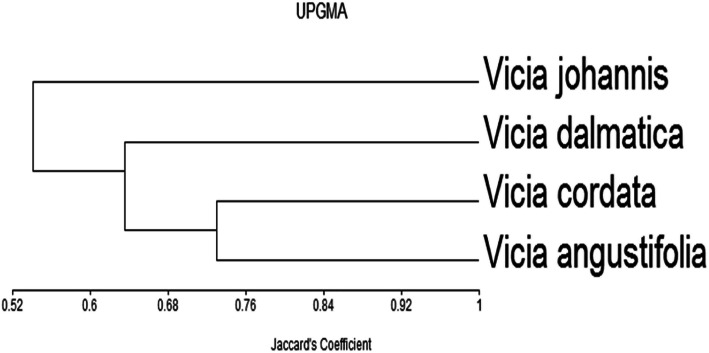
Dendrogram illustrating the relationships of the four *Vicia* species based on seed storage proteins, and as constructed by unweighted pair group arithmetic (UPGMA) and similarity matrices computed according to Jaccardʼs coefficient

The similarity indices among these species were estimated for each pair-wise group (Table 2 and Fig. 3). The highest similarity index (0.73) was recorded between *Vicia cordata* and *Vicia angustifolia*, while the lowest similarity index (0.51) was recorded between *Vicia johannis* and *Vicia angustifolia*. The dendrogram gave two main genetic clusters; the first cluster includes the species *Vicia johannis*, while the second cluster includes all other studied species. The second cluster was further divided into two sub-clusters; the first sub-cluster includes *Vicia dalmatica* only, while the second sub-cluster includes *Vicia cordata* and *Vicia angustifolia* (Fig. 3).

**Table 2:** Similarity matrix among studied *Vicia* species as computed according to Jaccardʼs coefficient as revealed by protein markers

***Vicia angustifolia***	1			
***Vicia cordata***	**0.73**	**1**		
***Vicia dalmatica***	**0.58**	**0.69**	**1**	
***Vicia johannis***	**0.51**	**0.55**	**0.56**	**1**
	***Vicia angustifolia***	***Vicia cordata***	***Vicia dalmatica***	***Vicia johannis***

## Discussion

Family Fabaceae is one of the world’s three largest families. It comprises approximately 650 genera. *Vicia* as one the Fabaceae genera is represented by more than 170 species in the world, and is distributed mainly from North America, Asia, and Europe to the moderate regions of South America and tropical Africa. The genus has economic importance species especially the faba bean (*V*. *faba*) as one of the most favourable grain legumes over all the world and *V*. *ervilia* as well, in addition to some minor food crops and a number of forage plants, such as *V*. *sativa*, and *V*. *angustifolia* and *V*. *cordata* have developed as weedy strains [2, 3]. *Vicia* species have a basic chromosome ranged from x = 5, 6 to 7 [4, 6, 7], and only six of them are polyploid [5].

Cytogenetic approaches introduced a powerful system for tracing the organization of DNA sequences along the chromosome. Fluorescence in situ hybridization (FISH) is the most valuable technique in cytogenetic field, it was established more than 40 years ago and has been used to explain all inquiries correlated to mutation, structure changing, and evolution of individual chromosomes and the entire genomes as well. FISH has served as an important tool for chromosome identification in many plant species The 45S rDNA loci consist of tandem arrays of repeating units of the 18S, 5.8S, and 25S rRNA genes, up to thousands of copies of the repeat units may be present in plants, in addition to the 5S rRNA gene repeats (which occurs independent of the 45S rDNA) which are located at one or more locus on chromosome set, and their distinctive sites along chromosomes are providing valuable markers for chromosome identification and offering advantages in comparing different accessions or species [11, 46–48].

Molecular cytogenetic approaches like florescence in situ hybridization (FISH) have been applied in different *Vicia* species [23, 24, 49–51]. Faba bean (*Vicia faba*) is the most extensively studied species via in situ hybridization tool to localize the distribution of repetitive sequences and 5S and 45S rDNA on its chromosomes [25, 52–57, 24]. studied FISH with multiple repeated DNA (four BamHI classes) isolated from *V*. *faba* and probes of 5S rDNA and 25S rDNA. The authors analysed the chromosomal and genetic relationship between faba bean (*Vicia faba*) and the closely related wild species (*V*. *johannis*). The chromosomal sites of 5S rDNA and 25S rRNA genes could discriminate *V*. *faba* from its closely related species. Applied FISH to the chromosomes showed the existence of BamHI family sequences spread in heterochromatin and euchromatin of the *V*. *faba*, *V*. *narbonensis*, *V*. *hyaeniscyamus*, *V*. *galilaea*, *V*. *johannis*, and *V*. *bithynica*.

Florescence in situ hybridization (FISH) has been applied on *Vicia* species; the study [24] was a trial to find out the physical maps of the 5S rDNA and 25S rDNA sites on the chromosomes of various taxa within genus *Vicia* (Narbonensis, Villosa, and Sativa), it was the only publication which discussed the positions of 5S rDNA and 45S rDNA by FISH in three species under the current study (*Vicia johannis*, *V*. *angustifolia*, and *V*. *cordata*). The current study observations were in agreement with the result obtained by [24] concerning the loci of 45S rDNA and 5S rDNA on the opposite arms of the smallest chromosome pair (no. 5) in *Vicia cordata* (2n = 10). On the other hand, the current investigation is partially disagreed with the study [24] concerning *Vicia johannis* (2n = 14), where the locus of 45S rDNA was on the shortest chromosome (no. 7) in both studies, while the locus of 5S rDNA on chromosome pairs no. 3 in their observation, but in the existing study is on chromosome pairs no. 6. Regarding *V*. *angustifolia*, the present study is disagreed with them, in their study the loci of 5S rDNA and 45S rDNA were on chromosome pairs no. 4 and 5, respectively, while in the current investigation are on chromosome pairs no. 3 and 6, respectively.

In the present double FISH experiment using 45S and 5S rDNA probes, the result has not shown any variation in the number of loci, where one 45S rDNA and one 5S rDNA gene loci were detected in the early metaphase chromosomes of each of the studied species, but the positions along the chromosomes were different, both *Vicia johannis* (2n = 14, section Faba) and *Vicia dalmatica* (2n = 12, section Cracca) exhibited the same interstitial 45S rDNA gene loci, while *Vicia angustifolia* (2n = 12, section Vicia) and *Vicia cordata* (2n = 10, section Vicia) have shown single large stretched 45S rDNA loci almost at the terminal region of the shortest chromosome.

Protein fractions in legumes are powerful candidates for improving many food stuffs, such as bakery, meat, and dairy products [58]. Sodium dodecyl sulfate polyacrylamide gel electrophoresis (SDS-PAGE) was used to investigate the seed storage protein patterns in many legumes including *Vicia* in the same study [59], who analysed the seed protein patterns of 47 accessions belonging to 11 species and four tribes of legumes including *Vicia*. Another study [60] isolated the protein samples from 15 legume species and cultivars (vetches, pea, white lupin, and field bean) and analysed them by SDS-PAGE electrophoresis of the seed storage proteins and estimated their molecular weight and quantify their relative quantities.

Despite this, there were many other evolutionary studies to clarify the relationships among *Vicia* species by determining the electrophoretic seed proteins patterns [51, 61–68]. It was noticeable that most of previous systematic classification and genetic relationship studies on genus *Vicia* using SDS-PAGE have been concentrated on faba bean [69–73], which indicates that SDS-PAGE was a useful tool for genetic diversity analysis of faba bean.

There was no previous investigation referred to the species under the present study concerning SDS-PAGE. In the current study, seed proteins of four *Vicia* species were analysed by SDS-PAGE to evaluate the genetic diversity among them. Eighty different bands of proteins were identified in these species. Depending on the obtained data in Table 2 which shows the similarity matrix among them as computed according to Jaccard coefficient from the SDS-PAGE, it could be concluded that *V*. *cordata* is similar to *V*. *angustifolia* and *V*. *dalmatica* by a percentage of 73 and 69%, respectively, and the most related species to *V*. *johannis* is *V*. *dalmatica* (~ 64%).

By combining the whole collected results in the present study, depending on double FISH of rDNA gene loci, SDS-PAGE of seed storage protein, in addition to the previously known taxonomical and chromosome number information regarding the species under the current study, the results could be summarized as follows: On one hand, the two species which belong to section Vicia, *V*. *cordata* (2n = 10) and *V*. *angustifolia* (2n = 12) are the most related to each other by a percentage of 73% and both have shown single large stretched 45S rDNA loci almost at the terminal region of the shortest chromosome. On the other hand *V*. *cordata* was similar to *V*. *dalmatica* by a percentage of 69%. From another side, *V*. *dalmatica* (section Cracca) was similar to *V*. *angustifolia* by a percentage of 58%, in the chromosome number (2n = 12) and almost in chromosome size as well, and it was observed that the most related species to *V*. *johannis* (section Faba, 2n = 14) is *V*. *dalmatica* (56%) and both exhibited the same large interstitial 45S rDNA gene loci.

Finally, according to these explanations, it could be concluded that the genetic relationship among the studied species is that *V*. *angustifolia* is more related to *V*. *cordata* then comes *V*. *dalmatica* and then *V*. *johannis* at a literal position from the other species.

## Conclusion

Cytogenetic approaches like FISH offer a powerful tool for detecting the organization of highly repeated DNA sequences (e.g., 45S and 5S rRNA genes) on the chromosome by using in situ hybridization of labelled probe sequences directly on the chromosomes. The 45S rRNA with the 5S rRNA genes (occurring as independent tandem arrays of repeating units) are located at one or more sites on the chromosome set, and their distinctive positions on the chromosomes afford useful markers for chromosome identification. The seed storage proteins are characterized by a high degree of polymorphism and limited environmental effect on their electrophoretic patterns; therefore, they have been successfully used as genetic markers for genetic diversity. Traditionally, SDS-PAGE has been used for protein separation and molecular weight determination as a qualitative tool for the analysis of seed legume in the breeding programme (e.g., nutritional qualities of bread and insect resistance) and for the purpose of varietal identification. The current results testified that FISH approach and storage protein profile have successfully reflected the relationships between the studied species.

## Data Availability

The authors declare that all generated and analysed data are included in the article. All plant materials (different *Vicia* species seeds) were identified and collected in the Institute of Plant Genetics and Crop Plant Research (IPK), Gatersleben, Germany.

## Abbreviations

FISH: Fluorescence in situ hybridization
SDS-PAGE: Sodium dodecyl sulfate-poly acrylamide gel electrophoretic
NORs: Nucleolar organizing regions
DAPI: 4′,6-Diamidin-2-phenylindol
MVSP: Multi-Variant Statistical Package
UPGMA: Unweighted pair group method with arithmetic
rDNA: Ribosomal DNA
